# The Push and Pull of Biomimicry in Construction: Identifying Key Drivers for Sustainable Transformation

**DOI:** 10.3390/biomimetics11030163

**Published:** 2026-03-01

**Authors:** Olusegun Aanuoluwapo Oguntona

**Affiliations:** Department of Built Environment, Faculty of Engineering, Built Environment and Information Technology, Walter Sisulu University, East London 5200, South Africa; ooguntona@wsu.ac.za

**Keywords:** biomimicry, built environment, nature, Push–Pull–Mooring, sustainable construction

## Abstract

The global construction industry is a primary driver of environmental degradation, resource depletion, and carbon emissions, necessitating an urgent transition toward sustainable practices. Biomimicry, the emulation of nature’s time-tested strategies, offers a transformative pathway for this shift, yet its systematic adoption remains inconsistent. This study utilises the Push–Pull–Mooring (PPM) framework to identify the critical drivers and contextual influencers of biomimicry adoption within the South African construction sector. A quantitative research approach was employed, involving a structured questionnaire survey of 104 diverse built environment professionals and subsequent analysis through Descriptive Statistics and Exploratory Factor Analysis (EFA). Descriptive results indicate that providing biomimicry education and training, increasing stakeholder awareness, and improving the availability of biomimetic technology are the highest-ranked drivers for sustainable transformation. EFA revealed a singular, dominant component termed the “systematic driver of biomimicry thinking”, which accounts for 54.2% of the total variance. The result emphasises the necessity of legal frameworks, policy monitoring, and government support. The findings conclude that while the Fourth Industrial Revolution provides the technological tools for bio-inspired innovation, a multi-layered approach combining institutional policy reforms with interdisciplinary education is essential to overcome traditional industry moorings. These insights offer a roadmap for stakeholders to leverage biomimicry as a cornerstone of resilient, regenerative and sustainable construction.

## 1. Introduction

The construction industry (CI) is a significant contributor to the global economy, accounting for 10–12% of the global economy [1]. It plays a vital role in socio-economic development by generating employment, upgrading infrastructure, and boosting economic activities [2]. However, the industry faces numerous challenges that impact its performance and outcomes. These challenges have therefore led to a global perception of the CI as the core sector responsible for environmental degradation, pollution, and other adverse impacts.

The construction sector is highly energy-intensive and has significant environmental impacts [3]. Construction activities pose serious health risks to workers and nearby residents, including respiratory problems, hearing impairment, and other conditions caused by dust, noise, and pollution [4]. The sector is a major consumer of natural resources, including building materials and energy, leading to resource depletion and significant ecological footprints [4,5]. Similarly, construction activities contribute to around 25% of air pollution, 40% of water pollution, and 50% of landfill waste, while the production of construction materials accounts for about 15% of all CO_2_ emissions [5,6]. The industry generates substantial construction waste, posing significant disposal challenges and environmental hazards [7]. The CI has been slow to integrate new technologies such as artificial intelligence (AI), robotics, and Industry 4.0 tools, which have the potential to enhance project efficiency, safety, and sustainability [8,9]. There are also issues of a lack of skilled workforce and high initial costs of technological tools, thereby hampering the efficiency and advancement of the CI [10,11]. Construction projects often underperform due to ineffective decision-making and project management, resulting in increased costs, delays, and defects [8]. The sector significantly contributes to greenhouse gas emissions, exacerbating climate change [12,13]. The adverse impacts highlighted the necessity of a shift towards sustainable construction practices.

Sustainable construction is a comprehensive approach that aims to minimise the environmental impact of building activities while promoting economic and social benefits. It integrates principles of sustainable development (SD) into the CI, focusing on the efficient use of resources and reducing negative impacts on human health and the environment [14,15,16]. Three major principles of sustainable construction are identified as environmental responsibility, social equity, and economic viability. These principles are aligned with the three pillars of SD as postulated when the term became popular. To therefore ensure sustainable construction is truly achieved, emphasis is placed on resource efficiency (efficient use of land, water, energy and materials), eco-friendly materials (use of renewable and recyclable resources), life cycle assessment of construction processes and materials, cost efficiency, health and well-being (improving health, social well-being and living conditions), and public participation in the planning and decision-making processes of construction projects [14,17,18,19,20,21,22].

Various sustainable construction practices (SCPs) encompass and integrate the principles of sustainable construction or the pillars of SD. Some of these SCPs focus on each principle, while a few address all three holistically. Concepts and practices related to sustainability in construction are often described using various terms. Terms or practices such as the precautionary principle, biophilia, ecological economics, eco-efficiency, the Natural Step, ecological rucksack, ecological footprint, Factor 4 and Factor 10, and biomimicry are used to describe the overarching concept of sustainability in the CI [23]. However, among these concepts, biomimicry stood out as one that embraces and integrates the three core pillars of SD. Biomimicry, as a nascent field, fosters transdisciplinary collaboration between biologists and professionals (e.g., designers, architects, medical scientists) to study and translate nature’s efficient strategies into sustainable solutions for the challenges of the built and human environment [24,25]. While the field of biomimicry has the potential to offer numerous innovative solutions inspired by nature and rooted in the concept of SD, it is important to identify ways of promoting the uptake of biomimicry for the sustainable transformation of the CI. Identifying these drivers will enhance practitioners’ and relevant stakeholders’ capacity to overcome the hindrances to the adoption and successful implementation of biomimicry for sustainable development.

## 2. Biomimicry in Construction

Biomimicry, the practice of emulating nature’s designs and processes to solve human challenges, is increasingly being recognised for its potential to revolutionise the construction industry (CI). In contrast to biomorphism, which is concerned with biological form, biomimicry prioritises the emulation of nature’s functions, systems, and processes. Its application has been witnessed in several areas within the CI, including material selection, structural design, and energy efficiency. For instance, biomimetic materials inspired by natural organisms and systems have been developed to enhance sustainability and resilience in construction projects [26,27,28]. These materials often mimic the properties of biological entities, such as the lightweight yet strong structure of bones or the self-cleaning surfaces of lotus leaves [29]. In structural design, biomimicry has led to the creation of innovative building forms and systems. Tensegrity structures, inspired by the natural balance of tension and compression found in biological systems, offer significant material and energy savings [30]. Additionally, biomimetic design frameworks have been proposed to guide architects in aligning their designs with suitable biological analogues, thereby optimising structural and environmental performance [26].

The adoption of biomimicry in construction offers numerous benefits, including enhanced sustainability, energy efficiency, and resilience. By emulating nature’s efficient use of resources, biomimetic designs can significantly reduce material consumption and waste [30]. For example, buildings with biomimetic skins inspired by natural thermal regulation mechanisms can achieve optimal thermal comfort and energy savings [31]. Moreover, biomimicry promotes the creation of green markets and services, protection of biodiversity, and conservation of natural resources [32]. These benefits align with global sustainability goals, such as the United Nations’ Sustainable Development Goal 11, which aims to make cities and human settlements inclusive, safe, resilient, and sustainable [33].

Despite its potential, implementing biomimicry in construction faces several challenges. One major barrier is the lack of knowledge and understanding of biomimetic principles among construction professionals [34]. Additionally, the traditional nature of the CI, coupled with outdated legislation and planning processes, hinders the adoption of innovative biomimetic solutions [34]. There are also technical challenges related to the scalability and practical application of biomimetic designs. For instance, while tensegrity structures offer theoretical benefits, their construction remains challenging, and most exist only as prototypes [30]. Furthermore, the perception of high risks and costs associated with biomimetic projects can deter investment and experimentation [34]. Other barriers to the adoption of biomimicry in construction include complexity in design and implementation, high initial costs, and limited knowledge [32,34].

Several case studies highlight the successful application of biomimicry in construction. For example, the use of snake-inspired facades in architecture has demonstrated various advantages, such as improved energy efficiency and aesthetic appeal [33]. Another notable example is the development of 3D-printed homes using biomimetic principles, which offer rapid, sustainable solutions to urbanisation [35]. In South Africa, biomimetic materials have been promoted to achieve sustainability in the CI, challenging stakeholders to adopt nature-inspired solutions [28]. These examples underscore the potential of biomimicry to drive innovation and sustainability in construction.

Biomimicry holds significant promise for transforming the CI by offering sustainable, efficient, and resilient solutions. By learning from nature, the CI can develop innovative materials and techniques that reduce environmental impact and enhance structural performance. While challenges remain, the growing interest and successful case studies demonstrate the potential of biomimicry to address some of the industry’s most pressing issues. Therefore, as the CI continues to evolve, the application of biomimicry principles holds the promise of achieving greater sustainability and efficiency in building practices, ultimately contributing to the development of resilient and environmentally friendly built environments. Hence, there is a need to identify the key drivers of biomimicry for the sustainable transformation of the CI.

## 3. Push–Pull–Mooring Framework in Biomimicry

The Push–Pull–Mooring (PPM) Framework is a theoretical construct used to understand switching behaviour across diverse contexts, such as technology adoption, consumer migration, and organisational change. Originating from the human migration theory, the PPM framework delineates three main categories of influences: push factors, pull factors, and mooring factors [36]. Push factors represent negative aspects of the current situation (such as dissatisfaction or risk) that motivate individuals to leave their current state [37]. Pull factors are positive attributes or benefits of an alternative that attract individuals to switch, including perceived usefulness, convenience, or superior service [38,39]. Mooring factors encompass personal, social, or contextual elements (such as habits, inertia, switching costs, or social norms) that moderate or impede the switching process [40,41,42]. Empirical studies consistently demonstrate that push and pull factors positively affect switching intentions, while mooring factors can inhibit or moderate these effects [37,38,39,40,41,42]. The PPM framework is valued for its nuanced integration of rational, emotional, and contextual drivers, offering robust insights into why individuals, groups, or organisations opt for change.

The CI is undergoing a paradigm shift driven by escalating environmental pressures, resource constraints, and the need for resilient built environment systems. Biomimicry, an approach that studies and emulates nature’s time-tested strategies, has emerged as a promising pathway for regenerative design and sustainable construction [26,43,44]. Despite its potential, the systematic adoption of this novel concept across the construction sector remains uneven. Hence, integrating the PPM framework offers a robust analytical lens for understanding the drivers of biomimicry transition, the attractors that facilitate uptake, and the institutional or technological moorings that mediate its adoption. Although originally applied to human migration and consumer behaviour [45,46], the PPM framework provides conceptual clarity for examining why it is imperative that the CI shift from conventional paradigms to nature-inspired innovation. The framework guides researchers and practitioners in identifying the drivers, attractors, and facilitators of biomimicry, ultimately promoting sustainable and innovative solutions inspired by nature [47,48]. Although interest in biomimicry as a sustainability strategy is increasing, there is limited empirical evidence regarding the factors influencing its adoption in the construction sector, especially within developing countries. This research seeks to address this gap by empirically identifying and organising the primary drivers of biomimicry adoption through the application of the Push–Pull–Mooring framework.

The application of the PPM framework within biomimicry, particularly to examine its utility for understanding adoption, innovation, and migration phenomena in bio-inspired applications, is imperative. This framework, commonly employed in social sciences to explain complex behavioural dynamics, offers a robust lens through which to analyse the drivers and inhibitors of biomimicry adoption, application and implementation [49]. Specifically, the ‘push’ factors in this context could include limitations of conventional design paradigms, such as resource depletion or environmental degradation, compelling a shift towards nature-inspired solutions. Conversely, ‘pull’ factors might consist of the inherent elegance, efficiency, and sustainability demonstrated by biological systems, attracting designers to nature-inspired approaches [50]. Furthermore, ‘mooring’ factors could encompass contextual elements that facilitate or impede the successful integration of biomimicry, such as organisational culture, technological infrastructure, or regulatory frameworks [51]. This comprehensive approach allows for a nuanced understanding of the forces shaping the adoption and diffusion of nature-inspired (biomimicry) innovations, bridging the gap between theoretical constructs and practical application. The increasing prominence of biomimicry as a research field, evidenced by a sevenfold increase in relevant indices since 2000, underscores the need for such a framework to elucidate its expanding influence and practical applications [52]. Therefore, it is important to examine the role of the PPM framework in biomimicry towards the sustainable transformation of the CI.

### 3.1. Push Factors: Pressures Driving Transformation in the Construction Industry

Table 1 summarises the push factors that collectively drive transformation in the CI, compelling it to adapt to new and changing economic, technological, environmental, and regulatory landscapes. The CI faces increasing globalisation, demanding larger, more complex projects delivered in shorter timeframes. This creates pressure from clients and governments to differentiate based on customer focus, product quality, and value creation [53]. Similarly, economic factors such as urbanisation and foreign direct investment (FDI) drive the need for green technology innovation and efficiency improvements in the construction sector [54].

The advent of Industry 4.0 technologies, including digital tools and automation, is also pushing the CI to adopt new approaches and skills to remain competitive [55]. The shift towards a digital economy requires adopting digital technologies to improve efficiency, productivity, and project outcomes. This includes the use of technologies such as Building Information Modeling (BIM), IoT, drones, and digital twins [56,57]. More notable is the significant push towards sustainable construction to meet environmental, social, and economic sustainability goals. This includes adopting sustainable technologies and practices to reduce the environmental impact of construction activities [54]. One of such practices or concepts is biomimicry, a nature-inspired paradigm revolutionising the construction sector and many others. Similarly, environmental legislation and public concern about sustainability are driving the adoption of sustainable technologies and practices in the CI [58].

Compliance with new conformity verification systems and regulatory requirements is another major pressure point, necessitating significant changes in CI practices and processes [59]. Institutional pressures, such as coercive, normative, and mimetic pressures from stakeholders, including government regulations and industry standards, are another significant driver of digital and sustainable transformation in the CI [60]. Also, the CI is traditionally conservative and resistant to change, making it difficult to transition to a sustainable state. However, overcoming this resistance is critical to adopting new technologies and practices [61]. Also, workforce challenges such as high turnover rates, job-demand pressures, and the need for new skills are pushing the industry to transform its workforce management and training practices [62]. Furthermore, increasing client demands for more flexible, efficient, and sustainable construction solutions are driving industry transformation [63]. Pressure to improve collaboration and communication among stakeholders, including supply chain partners, is also a significant driver of digital transformation [64] in the CIs. These push factors, among others, necessitate a drive toward transforming the CI in alignment with sustainable goals and focus.

**Table 1:** Summary of push factors driving transformation in the construction industry.

Push Factors	Description	Sources
Client and Stakeholder Expectations	Client demands, stakeholder collaboration	[53,63,64]
Organisational and Workforce	Resistance to change, workforce challenges	[61]
Institutional and Regulatory	Institutional pressures, regulatory compliance	[59,60]
Environmental and Sustainability	Sustainability goals, green technology innovation	[54,58,65]
Technological Advancements	Industry 4.0, digital transformation, new technologies	[55,56,57]
Economic and Competitive Pressures	Globalisation, market demands, economic development	[53,54]

### 3.2. Pull Factors: The Attractiveness of Biomimicry for Innovative Solutions

Biomimicry, the practice of emulating nature’s forms, designs, systems and processes to solve human challenges, offers several attractive pull factors for driving innovative solutions across various fields, including the CI. Biomimicry is applicable across industries such as construction, textiles, medicine, and technology. It offers solutions for sustainable construction practices, innovative textiles, and advanced medical treatments [32,66,67]. Notably, biomimicry is widely known for resource optimisation and efficiency. Biomimicry can significantly reduce resource consumption. For instance, a biomimicry-driven project by GOJO Industries produced twice the intellectual property and energy savings with just one-sixth the resource commitment required by traditional methods [68]. Nature-inspired designs, innovations and solutions, such as those used in construction and textiles, often lead to energy-efficient outcomes. For example, bio-inspired materials in architecture and construction can reduce energy demands by facilitating environmental adaptation [66,69].

Biomimicry also offers environmental and sustainability benefits. Biomimicry promotes environmentally sustainable innovation by emulating nature’s time-tested strategies, forms and processes. This approach has been demonstrated to protect biodiversity, conserve natural resources, and foster the development of green markets and services [32,70]. Biomimicry approaches in material science and manufacturing can also enhance sustainability by reducing environmental impact and improving resource efficiency [67,71]. Biomimicry often leads to the development of high-performance materials and systems. Examples include self-repairing textiles, superhydrophobic surfaces, and energy-efficient building designs inspired by natural structures [66,72]. Similarly, biomimetic applications have led to novel solutions across various fields, including adaptive locomotion for lunar exploration, bio-inspired robotics, and advanced materials for extreme environments [73].

The interdisciplinary nature of biomimicry fosters collaboration among designers, engineers, and scientists, leading to more holistic and innovative solutions [69,74]. Companies or organisations adopting biomimicry can gain a strategic advantage by developing unique, sustainable products that meet the growing demand for environmentally friendly solutions [75]. Similarly, biomimicry can help businesses differentiate themselves in the market by offering innovative and sustainable products that stand out from conventional offerings [68,71]. This is because biomimicry can accelerate front-end innovation. The case study of GOJO Industries demonstrated that biomimicry could double the speed of innovation compared to conventional methods [68]. Biomimicry also provides creative solutions to complex problems in design, transformation, and organisation, making it a valuable approach for businesses [76]. Biomimicry’s attractiveness lies in its ability to drive efficient, sustainable, and innovative solutions across various sectors. By emulating nature’s strategies, biomimicry not only enhances performance and functionality but also promotes environmental sustainability and provides a competitive edge in the green market. The interdisciplinary and resource-efficient nature of biomimicry makes it a highly promising approach for addressing modern technological and environmental challenges.

### 3.3. Mooring Factors: Moderators of Biomimicry Adoption in Construction

The adoption of biomimicry in construction is moderated by a combination of organisational, economic, regulatory, collaborative, market, and technological factors. Understanding these mooring factors can help stakeholders identify barriers and enablers to effectively integrate biomimetic practices into construction projects, ultimately leading to more sustainable and innovative outcomes. Biomimicry in construction encounters multiple challenges, including technical barriers such as limited design expertise and a lack of established standards, financial concerns related to perceived higher costs and risks, and organisational issues such as resistance to change and fragmented project delivery. Research on biomimicry-based projects indicates that successful implementation depends on early integration of biomimetic principles, interdisciplinary collaboration, and the presence of supportive governance structures. A significant barrier to the adoption of biomimicry is the lack of knowledge and awareness among construction professionals about its principles and applications [77,78,79]. This gap necessitates targeted educational programmes and awareness campaigns to bridge the knowledge gap. The level of awareness and understanding of biomimicry among stakeholders, including architects, engineers, and contractors, plays a crucial role in its adoption. Implementing educational initiatives to enhance knowledge and familiarity with biomimicry concepts is vital [78,79,80].

The presence or absence of supportive regulatory frameworks significantly influences the adoption of biomimicry. Regulatory measures can either facilitate or hinder the integration of biomimetic strategies in construction practices [24,79]. There is also a need for policy reforms to create an enabling environment for the adoption of biomimicry, including incentives and guidelines that promote sustainable construction practices [24,25,79]. Stakeholders’ perceptions of risks associated with adopting new and innovative biomimetic solutions can be a deterrent. These risks include uncertainties about performance, cost implications, and long-term benefits [24,79]. Having clear-cut guidelines and well-communicated potential benefits of adopting biomimicry will help overcome risk perceptions. The initial costs and economic feasibility of bio-inspired solutions are other critical factors. Stakeholders need clear cost–benefit analyses to justify investment in biomimicry [24,25,81].

The efficient use of resources and the environmental benefits of biomimicry are significant motivators. Biomimicry can lead to the conservation of natural resources and the protection of biodiversity [24,25,70]. Aligning biomimicry adoption with broader sustainability goals, such as reducing greenhouse gas emissions and improving energy efficiency, can drive its acceptance [77,82,83]. Institutional factors, such as support from professional bodies and industry associations, also facilitate biomimicry adoption [82]. The involvement and perspectives of various stakeholders, including construction workers, supervisors, regulatory bodies, and corporate management, are very crucial for the successful adoption of biomimicry [81]. Addressing these moderators through targeted educational initiatives, supportive policies, risk mitigation strategies, and stakeholder engagement can facilitate the integration of biomimicry and promote sustainable construction practices.

## 4. Research Methodology

The quantitative research approach was employed in this study to determine the key drivers of biomimicry for the sustainable transformation of the CI. Quantitative research is described as a formal, objective, rigorous, and systematic process employed to describe, investigate, and test relationships, causes, effects, and interactions among different variables [84]. It serves as a survey method for collecting data from study participants (the sample) through objective self-reporting, in which subjects respond to inquiries posed by the researcher [84,85]. Although biomimicry is still an emerging concept in the construction industry, the objective of this study was to identify, rank, and structure the key drivers influencing its adoption at an industry-wide level, rather than to conduct a phenomenological exploration. A quantitative survey was selected because it facilitates the aggregation of perceptions from a broad professional population and supports statistical analyses such as descriptive ranking and exploratory factor analysis. This methodological approach is consistent with previous research examining drivers of sustainability-oriented innovation and behavioural transitions in construction and built environment studies. The descriptive survey was adopted in this research study because it provides a concise account of the attributes (i.e., behaviours, opinions, beliefs, abilities, and knowledge) of a specific individual, group, or situation. The method was preferred to achieve the objective of this study, which is to identify the key drivers of biomimicry for the sustainable transformation of the CI in South Africa.

The study was carried out in the Gauteng and Western Cape provinces of South Africa. Gauteng and Western Cape Provinces were selected because they are jointly the largest contributors to South Africa’s GDP and home to major construction activities, professional bodies, and stakeholders. The provinces were also selected because of the high concentration of green building projects and because they are home to major green collaborative networks and stakeholders (i.e., the Green Building Council of South Africa and the Sustainability Institute). A non-probability purposive sampling strategy was employed, focusing on built environment professionals directly involved in construction decision-making and sustainability practices. Due to the emerging status of biomimicry within the South African construction industry, purposive sampling was considered suitable to ensure respondents had adequate professional experience to assess biomimicry-related drivers effectively. While the sampling approach does not achieve full statistical representativeness of the broader construction workforce, the sample remains analytically representative of professionals most likely to influence or implement biomimicry-based innovations. Potential biases, such as self-selection and professional affiliation bias, are recognised. These biases are common in innovation adoption studies within professionalised industries and do not compromise the validity of the exploratory analysis. Architects, quantity surveyors, engineers, construction managers, project managers, and biomimicry specialists/professionals in the South African construction industry constitute the target population (respondents) for this study. These respondents are practising and duly registered construction professionals in South Africa.

Data were gathered for this research through a well-structured questionnaire distributed to the study participants (respondents) by the researcher. The survey instrument was constructed following a comprehensive review of previous research on biomimicry adoption, sustainable construction practices, and innovation diffusion in the built environment. Foundational sources guided the identification of potential drivers, drawing from literature addressing the benefits and barriers of biomimicry, sustainability-driven innovation, and technology adoption in construction. The identified drivers were systematically mapped to the Push–Pull–Mooring (PPM) framework. Push-related items represented pressures that motivate a shift away from conventional construction practices, including environmental degradation, regulatory requirements, and technological inefficiencies. Pull-related items encompassed the perceived benefits of biomimicry, such as innovation potential, resource efficiency, enhanced performance, and improved sustainability outcomes. Mooring-related items encompassed contextual and moderating factors affecting adoption, including legal and regulatory frameworks, policy monitoring, institutional support, the availability of standards, the affordability of biomimetic solutions, and certification mechanisms. To improve content validity and clarity, the draft questionnaire was reviewed by a group of experienced built environment professionals knowledgeable in sustainability and biomimicry. A pilot test evaluated the clarity, relevance, and alignment of items with the study objectives. Feedback from this process led to minor revisions in item wording and structure before the main survey administration. A total of 120 questionnaires were administered to the respondents, but only 104 were completed, received and used for the analysis. The study adopted a closed-ended questionnaire because it is easier to administer, analyse, and determine. The questionnaire was designed using a 5-point Likert scale (agreement scale), with 1 indicating the lowest response and 5 indicating the highest response. The respondents were also assured of the anonymity of their responses.

Descriptive and inferential statistical techniques were applied, and data analysis was performed using the Statistical Package for the Social Sciences (SPSS) software (version 21). To analyse the respondents’ demographic data, percentages were used. The final set of survey items, which were aligned with the PPM constructs, underwent descriptive analysis and exploratory factor analysis to empirically identify the underlying dimensions influencing biomimicry adoption. The dataset was assessed using the mean score (MS), standard deviation (SD), and factor analysis (exploratory factor analysis). The mean scores of the results were used to rank the identified key drivers of biomimicry for the sustainable transformation of the CI. As one of the most commonly reported quantitative methodologies in the social sciences, exploratory factor analysis (EFA) was employed to identify underlying latent variables responsible for the observed covariance among manifest variables [86,87]. During factor extraction, the shared variance of a variable is separated from its unique and error variances to uncover the inherent factor structure, meaning the result reflects only the shared variation. The Cronbach’s alpha test was performed to ensure the validity and reliability of the survey instrument (questionnaire). The test yielded a Cronbach’s alpha of 0.838, deemed acceptable and indicative of high reliability. Beyond establishing internal consistency reliability, content validity was addressed through an extensive review of literature on biomimicry, sustainable construction, and innovation adoption, complemented by expert evaluation of the survey instrument. This approach ensured that the questionnaire items were relevant, unambiguous, and effectively represented the conceptual domains under investigation. This methodology aligns with exploratory investigations of emergent phenomena, in which theory development and construct refinement are prioritised before confirmatory testing.

## 5. Presentation of Results

This section presents the background information of the respondents, the mean score, standard deviation, rank and exploratory factor analysis (EFA) of the key drivers of biomimicry for the sustainable transformation of the CI.

### 5.1. Demographic Information of the Respondents

The respondents’ demographic information is presented in Table 2. According to the table, 53.8% of respondents are male, and 46.2% are female, indicating progress in the South African government’s efforts to bridge the gender gap in the built environment, which is traditionally male-dominated. Table 2 further revealed that 24% of the respondents are biomimicry professionals/experts, 19.2% are quantity surveyors, 18.3% are architects, 15.4% are engineers, and 11.5% are both construction managers and project managers. Regarding the respondents’ years of professional practice/experience, 53.8% had 1–5 years of experience, 20.2% had 6–10 years of experience, 15.4% had 11–15 years of experience, 4.8% had 16–20 years of experience, and 5.8% had more than 20 years of experience.

### 5.2. Drivers of Biomimicry for Sustainable Transformation of the Construction Industry

Table 3 shows the respondents’ rankings of the drivers of biomimicry for the sustainable transformation of the CI. The table shows that ‘providing biomimicry education and training’ was ranked first with a mean score of 4.69 and standard deviation of 0.464; ‘increasing client and stakeholder’s awareness’ was ranked second with a mean score of 4.51 and standard deviation of 0.607; ‘improving availability of biomimetic technology’ was ranked third with a mean score of 4.45 and standard deviation of 0.500; ‘improving availability of biomimetic materials’ was ranked fourth with a mean score of 4.39 and standard deviation of 0.645; and ‘improved affordability of biomimetic materials’ was ranked fifth with a mean score of 4.38 and standard deviation of 0.685. The lower part of the table shows that ‘developing a policy monitoring system’ with a mean score of 3.96 and standard deviation of 1.004 was ranked twelfth; ‘developing a legal and regulatory framework’ with a mean score of 3.91 and standard deviation of 1.158 was ranked thirteenth; and ‘providing motivation and commitment (self and corporate)’ was ranked fourteenth with a mean score of 3.90 and standard deviation of 0.807.

An EFA was conducted without specifying the number of factors in advance, thereby permitting the data structure to emerge naturally. The initial extraction identified a single dominant factor with an eigenvalue above the recommended threshold, accounting for a significant proportion of the total variance. Item loadings on this factor were robust and surpassed established cut-off values, demonstrating internal consistency. Although multi-factor solutions were explored, they were not retained because of low secondary eigenvalues, substantial cross-loadings, and insufficient conceptual distinction among extracted factors. Retaining these solutions would have undermined both statistical validity and theoretical clarity. Therefore, the one-factor solution was identified as the most parsimonious and empirically justified representation of the data. As shown in Table 4, the information presented confirms that the data used for the factor analysis is appropriate for this type of statistical procedure. The KMO measure of sampling adequacy achieved a high value of 0.828, exceeding the recommended minimum value of 0.6 (60.0%) by Kaiser [88]. Bartlett’s Test of Sphericity was also statistically significant (*p* < 0.05), thus supporting the factorability of the correlation matrix. From Table 4, Bartlett’s Test of sphericity shows an approximate Chi-Square value of 284.284 at a degree of freedom (df) of 21, with an acquired statistical significance of 0.000 (*p* < 0.05). These results support the factorability of the correlation matrix for the construct’s variables, validating the use of factor analysis on the provided dataset.

**Figure 1:**
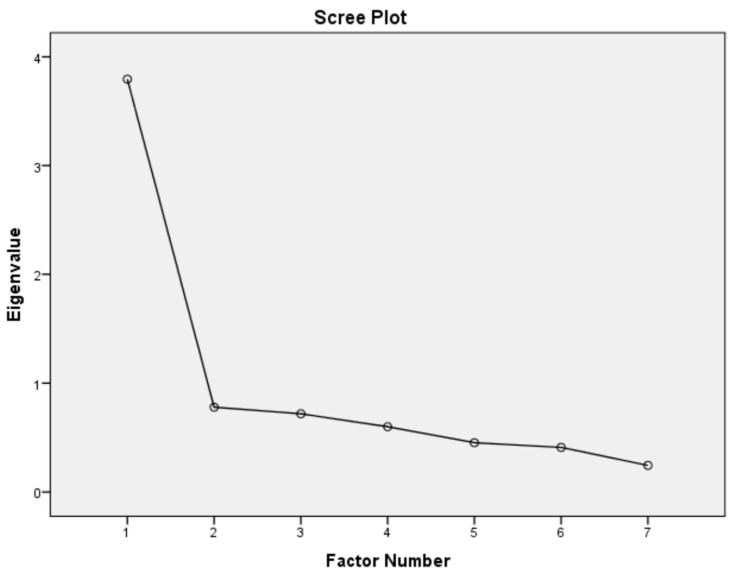
Scree plot of biomimicry drivers for sustainable transformation in the CI.

The Total Variance explained with the Principal Axis Factoring (PAF) extraction method for the biomimicry drivers for sustainable transformation in the CI is presented in Table 5. The table shows that one (1) component has an initial eigenvalue exceeding 1 for the rotated loadings. The total variance explained by the extracted factor (component 1) is 54.204%. Thus, the final PAF statistics and the extracted factors accounted for approximately 54% of the cumulative variance. This value meets the minimum acceptable threshold of more than 40%, which is considered sufficient for factor extraction, supporting the validity of the retention in this study [89]. The scree plot presented in Figure 1 also revealed the excluded factors by indicating the cut-off point at which the eigenvalues levelled off.

The data were subjected to PFA (with varimax rotation). The eigenvalue was set to a conventional high value of 1.0. Table 6 shows the rotated component matrix of the biomimicry drivers for sustainable transformation in the CI. The rotation converged in relation to the initial eigenvalues of 1 after seven (7) iterations. As shown in Table 6, only one (1) factor with eigenvalues exceeding 1.0 was extracted. The designated matrix segments pinpoint biomimicry drivers with negligible deviation from the primary eigenvalue. Based on the correlation matrix, several factors surpassed the 0.500 coefficient benchmark. During the purification process, variables that failed to meet the 0.500 cross-loading criterion were removed, and the remaining items were aggregated based on their shared variance. From using PAF with varimax rotation during the conduct of an EFA, seven (7) items met loading and communality thresholds and were retained. These highlighted values signify the primary loadings essential for establishing the construct clusters.

## 6. Discussion of Findings

The empirical findings were interpreted using the Push–Pull–Mooring (PPM) framework to elucidate how various categories of drivers influence biomimicry adoption in the South African construction industry. Push factors were identified as sources of dissatisfaction with conventional construction practices, such as environmental degradation, resource inefficiency, climate-related pressures, and increasing sustainability expectations. These factors motivate a departure from traditional approaches. Pull factors were associated with the perceived benefits of biomimicry, including enhanced building performance, improved resource efficiency, increased innovation, and contributions to long-term sustainability. These advantages position biomimicry as a viable alternative to conventional design and construction. Mooring factors were linked to the institutional and contextual environment, encompassing regulatory frameworks, the availability of standards and certification mechanisms, policy monitoring, affordability, and professional capacity. While these factors do not independently drive adoption, they shape how push and pull influences are translated into implementation decisions.

### 6.1. Discussion of Findings: Descriptive Analysis

Several factors drive the adoption, incorporation, and use of biomimicry to optimise sustainability in the CI. Empirical findings indicated that providing biomimicry education and training, increasing clients’ and stakeholders’ awareness, and improving the availability of biomimetic technology are ranked as the top three drivers of biomimicry thinking in the South African CI. Owing to its significant benefits and potential, biomimicry has been integrated into higher education, particularly in engineering and design curricula. For example, universities in the US have developed interdisciplinary programmes that combine biology, design, engineering, and business to advance biomimicry research [90]. Similarly, a biomimicry module for engineering undergraduates in India showed significant improvement in students’ problem-solving skills and engagement [91]. Also, organisations like Great Lakes Biomimicry and Biomimicry 3.8 offer professional training to make biomimicry more accessible [90]. These trainings help professionals integrate biomimicry principles into their work, fostering innovation and sustainability.

Awareness of biomimicry across sectors, such as construction and urban design, is also growing. The practice of biomimicry is recognised for its potential to enhance sustainability and resilience in these sectors [70,92]. Increasing the general public awareness is therefore crucial for the adoption of biomimicry. Interestingly, studies have shown that educational resources can significantly enhance public understanding and interest in biomimicry, thereby driving future research and applications [93]. Studies emphasised that increased awareness and targeted education are essential to overcoming attitudinal, institutional, and regulatory barriers that hinder the successful integration of biomimicry. This conclusion aligns with the findings of the current study and further reinforces the significant potential of biomimicry to optimise sustainability in the CI.

Technological advances, which are the signature characteristics of the present fourth industrial revolution (4IR), also provide a significant opportunity to apply biomimicry to drive sustainable transformation of the CI. Advances in technology, such as nanotechnology, artificial intelligence (AI), unmanned aerial vehicles (UAVs) and robotics, have expanded the possibilities for biomimicry. Innovative solutions, such as the gecko-inspired adhesives and spider silk synthesis, demonstrate the growing sophistication of biomimetic applications in sectors such as the CI [94]. Another key factor in driving the application of biomimicry for a sustainable CI is multi-disciplinary and interdisciplinary collaboration. Effective and holistic biomimicry often requires collaboration across disciplines. For the advancement of biomimicry, collaboration across various disciplines and sectors is essential. Studies highlight the need for better methods to encourage cooperation among biologists, designers, engineers, and other stakeholders to fully leverage biomimetic innovations. Organisations like BIOKON International focus on building integrative networks to promote biomimetic innovations [95]. Similarly, sharing knowledge and best practices can help overcome challenges that hinder its application and leverage the transformative power of biomimicry to promote sustainable practices [70].

Government support through policy-making is crucial for the adoption and development of biomimetic materials. Policies that promote sustainable practices and incentivise innovation can significantly drive the biomimicry field [82,96]. Similarly, the development of international standards, such as those issued by ISO, can help establish a common vocabulary and framework, facilitating the adoption and commercialisation of biomimetic materials [97]. Companies are increasingly viewing environmental sustainability as a strategic driver of innovation and competitive advantage. This shift in perception is prompting a demand for sustainable design tools, including biomimicry [75]. The intrinsic motivation of R&D professionals, along with the strategic integration of biomimicry tools into existing workflows, can enhance creative output and streamline its integration into corporate settings [75]. These assertions strongly align with the findings of this study, further confirming that the factors revealed in Table 3, when embraced, will ensure that biomimicry, as a novel concept, sustainably transforms the CI. By promoting awareness, training, workshops, and education, biomimicry will be widely adopted and implemented.

### 6.2. Discussion of Findings: Clustered Factors from Exploratory Factor Analysis

The identification of a single dominant factor indicates that, within the context of biomimicry adoption in the South African construction industry, push, pull, and mooring influences are not regarded as distinct or sequential forces. Rather, these influences operate as an integrated decision-making construct shaped by concurrent environmental pressures, perceived innovation advantages, and enabling contextual conditions. This result broadens the application of the Push–Pull–Mooring framework by demonstrating that, in emergent sustainability transitions, its components may converge into a unified systemic driver rather than persist as independent dimensions. Biomimicry inherently integrates ecological performance, innovation, and institutional readiness, which may account for the observed empirical convergence. The one-factor solution thus reflects the holistic, systems-oriented nature of biomimicry-based sustainability transitions in construction.

From the exploratory factor analysis (EFA), the clustered factor provides valuable insights into the factors that drive the adoption, incorporation, and use of biomimicry to optimise sustainability in the CI. Principal axis factoring revealed the presence of one (1) factor with an eigenvalue above 1, as shown in Table 6. Based on the examination of the inherent relationships among the variables under the extracted factor, the following interpretation was made. With only one factor extracted, the cluster was termed “systematic driver of biomimicry thinking for sustainable construction”. The name for this factor was derived from a close examination of the variables within it. The constituent indicators of the extracted factor are explained next, together with a detailed description of the nature of each.

#### Cluster 1: Systematic Driver of Biomimicry Thinking for Sustainable Construction

As presented in Table 6, the seven (7) extracted drivers for Cluster 1 were developing a legal and regulatory framework (78.2%), developing a policy monitoring system (76.6%), improving government support and intervention (75.2%), improving availability of biomimetic framework and measurement standard (67.2%), improving availability of biomimetic technology (63.7%), improving affordability of biomimetic materials (59.8%), and providing biomimicry innovation and certification (55.6%). The number in parentheses indicates the respective factor loadings. This cluster accounted for 54.2 percent of the variance. This component is quite imperative for an effective and efficient application of biomimicry thinking towards the sustainable transformation of the CI. The constituting drivers in this cluster are also significant at the form, process, and ecosystem levels, as well as in the problem-based and solution-based approaches to holistic biomimicry application.

A multi-layered systematic approach is essential for embedding biomimicry into sustainable construction practices. Embracing a comprehensive legal and regulatory framework is crucial to overcoming barriers to the adoption of biomimicry for sustainable construction. Regulatory frameworks can help overcome obstacles to biomimicry adoption by setting clear standards and guidelines. Similarly, regulatory frameworks can mandate educational programmes to improve awareness among construction professionals. For example, in Nigeria, regulatory challenges are a significant barrier to biomimicry adoption, and implementing regulatory measures is recognised as a solution to address these challenges [98]. Similarly, in the United Arab Emirates (UAE), regulatory factors significantly influence stakeholder perceptions and intentions to adopt biomimicry. Policy reforms are necessary to facilitate the integration of biomimicry and support broader sustainability agendas [79]. Considering that resistance to change is a major barrier to the uptake of novel concepts, especially among professionals, policies backed by legislative frameworks, acts and relevant professional bodies are imperative. Improving professional practices through regulatory measures can ensure that construction professionals are well-versed in biomimicry principles and their applications [98]. For the potential benefits of biomimicry to be fully realised in the sustainable transformation of the CI, the cooperation and support of stakeholders (governmental, non-governmental, etc) is crucial.

Effective policies can help overcome barriers to the adoption of biomimetic technologies, such as high costs and lack of awareness among stakeholders [78,99]. By providing regulatory backing and incentives, governments can encourage the integration of biomimetic solutions in construction projects. Clear legal frameworks can guide the adoption of biomimetic and relevant digital technologies that support biomimicry, ensuring they are used effectively to promote sustainability in the CI [100]. Establishing clear guidelines and standards for biomimetic technologies can ensure their uptake and reliable implementation across the sector [101,102]. This can include developing evaluation frameworks and performance metrics to assess the effectiveness of these technologies [103]. Encouraging collaboration between academia, industry, and government can foster innovation and the development of new biomimetic applications [104,105]. Policies that support research and development initiatives can lead to breakthroughs in biomimetic technology and its practical applications in addressing unique sustainability challenges in the construction sector. Similarly, providing incentives for the use of biomimetic technologies that enhance sustainability can drive their adoption in the industry [78,106]. Such incentives may include tax breaks, subsidies and grants for construction projects that incorporate biomimetic solutions and technologies.

Overall, these drivers authenticate the disruptive role of biomimicry to effectively transform the construction sector sustainably. Not only do these drivers showcase the potential benefits of biomimicry, but they also address barriers to adoption, enhance awareness and education, support sustainable practices, encourage innovation and collaboration, and align with global sustainability goals. These factors also collectively create an environment conducive to innovation and sustainability in the CI. By implementing targeted regulatory measures and policy reforms, the construction industry can fully leverage the transformative potential of biomimicry to achieve sustainability and resilience.

## 7. Conclusions and Recommendations

The findings of this study underscore a pivotal shift in the architectural, engineering, and construction (AEC) sector, where the regenerative potential of biomimicry is increasingly challenging the traditional, linear “take–make–waste” model. By applying the Push–Pull–Mooring (PPM) framework, this research study has elucidated that the transition to sustainable construction is not merely a technical challenge but a complex behavioural migration. The “Push” factors, namely the dire environmental consequences of conventional construction and the depletion of virgin materials, have created a necessary urgency. However, analysis reveals that the “Pull” of biomimicry, characterised by its promise of resource efficiency, zero-waste systems, and high-performance materials such as self-healing concrete or thermal-regulating facades, acts as a powerful attractor for forward-thinking construction professionals. Accordingly, the Push–Pull–Mooring framework is utilised in this study as an interpretive lens rather than as a fixed factorial structure, thereby enabling empirical findings to inform and refine theoretical understanding. This research study successfully mapped these dynamics, providing a robust theoretical foundation for understanding how nature-inspired innovation can move from a niche architectural concept to a mainstream industrial standard, ultimately aligning human-built environments with the functional excellence of biological ecosystems.

The findings underscore the necessity for more coherent and enforceable sustainability policies within the South African construction sector, specifically those that explicitly recognise biomimicry and nature-based design approaches. Although national frameworks, including green building standards and climate commitments, are in place, their implementation as enforceable construction practices remains inconsistent. Empirically, data from the South African construction industry highlight a critical hierarchy of drivers that must be addressed to catalyse this transformation. The highest-ranked driver, the provision of biomimicry education and training, identifies a significant knowledge gap that currently acts as a formidable “Mooring” factor. The results indicate that while there is a burgeoning interest in sustainable transformation, the lack of standardised curricula and professional certification in nature-inspired design prevents practitioners from moving beyond the “Push” of environmental regulations toward the “Pull” of innovative application. The ranking of stakeholder awareness and the availability of biomimetic technology as subsequent top drivers suggests that the sector is currently in a state of unconscious incompetence regarding nature-inspired solutions. For biomimicry to achieve systemic adoption, the industry must move beyond aesthetic imitation (biomorphism) toward deep functional emulation, which requires a fundamental retooling of professional skill sets through interdisciplinary collaboration among biologists, scientists, designers, engineers, etc.

The Exploratory Factor Analysis (EFA) conducted in this research further reveals a “systematic driver of biomimicry thinking”, which accounts for over half of the total variance in the study. This component emphasises that individual enthusiasm for green design is insufficient without a supportive institutional scaffolding. The findings point to the need for integrated legal frameworks, rigorous policy monitoring, and direct government support to offset the “Mooring” effects of high initial costs and perceived risks associated with novel technologies. As we stand on the precipice of the Fourth Industrial Revolution (4IR), tools such as 3D printing, generative design, and synthetic biology offer the technical capability to manifest complex biological geometries and processes in the built environment. However, the EFA results suggest that these technological “Pulls” will only gain traction if they are underpinned by international standards (ISO) and economic incentives that reward long-term ecological performance over short-term capital expenditure.

Ultimately, the path forward for sustainable transformation in construction lies in the deliberate synchronisation of ecological intelligence and industrial capability. This study concludes that biomimicry is not just a tool for sustainability, but a survival strategy for an industry facing unprecedented environmental volatility. To bridge the gap between nature’s inspiration and construction reality, the sector must foster an ecosystem of innovation where policy serves as the catalyst, education as the bridge, and technology as the medium. Regulatory bodies and professional councils have a critical role in integrating biomimicry principles into building codes, procurement guidelines, and professional accreditation requirements. Additionally, targeted incentives, public-sector demonstration projects, and skills development programmes are necessary to mitigate perceived risks and cost barriers associated with biomimicry adoption, particularly in public infrastructure and housing projects. Consequently, the findings are generalisable primarily at the conceptual and theoretical levels. This is especially relevant for elucidating the underlying mechanisms that drive biomimicry adoption within the construction sector, as interpreted through the Push–Pull–Mooring framework. However, these findings should not be construed as statistically generalisable outcomes representative of the entire construction industry.

## 8. Limitations and Future Research Directions

Several limitations should be acknowledged in this study. First, the use of a non-probability sampling approach restricts the statistical generalisability of findings beyond the surveyed professional cohort. Second, the exploratory analysis and reliance on self-reported perceptions may not comprehensively capture project-specific implementation dynamics. Third, although the Push–Pull–Mooring framework served as a valuable interpretive lens, confirmatory validation of the identified factor structure was not undertaken. These limitations highlight the need for future research employing mixed-methods designs, larger samples, and confirmatory modelling techniques. Although this study utilised a quantitative survey to identify and structure the key drivers of biomimicry adoption, incorporating qualitative insights would enhance understanding of context-specific challenges and practitioner experiences.

Future research should consider mixed-methods approaches, such as interviews or case-based investigations, to provide deeper interpretive insights and to validate the quantitative patterns observed in this study. Future research should also focus on the longitudinal performance of biomimetic buildings to provide the empirical proof of concept that cautious stakeholders require. By embracing the lessons of the natural world, which have evolved over billions of years for resilience and efficiency, the CI can grow from a consumer of the planet’s health into a vital contributor to its restoration. This transition, though complex, is the only viable route toward a truly sustainable and regenerative global infrastructure. Construct validity for the study was examined using an exploratory approach. In line with the study’s aim to identify and structure the key drivers of biomimicry adoption, Exploratory Factor Analysis (EFA) was utilised to reveal latent dimensions within the data. As a result, confirmatory assessments of construct validity were not performed at this stage. Future research should therefore employ confirmatory factor analysis or structural equation modelling to further validate the factor structure identified in this study.

## Figures and Tables

**Table 2:** Demographics of the respondents.

Factors	Variables	Percentages (%)
Gender	Female	46.2
	Male	53.8
	Total	100
Professional Qualification	Biomimicry professionals	24.0
	Construction managers	11.5
	Project managers	11.5
	Engineers	15.4
	Quantity surveyors	19.2
	Architects	18.3
	Total	100
Years of Experience	1–5 years	53.8
	6–10 years	20.2
	11–15 years	15.4
	16–20 years	4.8
	20 years and above	5.8
	Total	100
Sectoral Affiliation	Contracting firm/sector	17.3
	Governmental firm/sector	24.0
	Consulting firm/sector	35.6
	Private/non-governmental organisation	23.1
	Total	100
Construction Projects Currently Handled	None	17.3
	1–2 projects	51.0
	3–4 projects	19.2
	5–6 projects	3.8
	7–8 projects	2.9
	More than 8 projects	5.8
	Total	100

**Table 3:** Ranking of biomimicry drivers for sustainable transformation in the construction industry.

Drivers	MS	SD	Rank
Providing biomimicry education and training	4.69	0.464	1
Increasing client and stakeholders’ awareness	4.51	0.607	2
Improving availability of biomimetic technology	4.45	0.500	3
Improving availability of biomimetic materials	4.39	0.645	4
Improved affordability of biomimetic materials	4.38	0.685	5
Increasing client demand	4.35	0.498	6
Providing economic incentives	4.30	0.652	7
Improving multi-disciplinary collaboration	4.19	0.712	8
Improving government support and intervention	4.18	0.983	9
Improving availability of biomimetic framework and measurement standard	4.11	0.787	10
Providing biomimicry innovation and certification	4.05	0.989	11
Developing a policy monitoring system	3.96	1.004	12
Developing a legal and regulatory framework	3.91	1.158	13
Providing motivation and commitment (self and corporate)	3.90	0.807	14

**Table 4:** KMO and Bartlett’s test on biomimicry drivers for sustainable transformation in the CI.

Kaiser–Meyer Measure of Sampling Adequacy		0.828
Bartlett’s Test of Sphericity	Approx. Chi-Square	284.284
	df	21
	Sig.	0.000

**Table 5:** Total variance explained on biomimicry drivers for sustainable transformation in the CI.

Variables	Initial Eigenvalues			Rotation Sums of Squared Loadings		
	Total	% of Variance	Cumulative %	Total	% of Variance	Cumulative %
1	3.794	54.204	54.204	3.794	54.204	54.204
2	0.779	11.131	65.335	0.779	11.131	65.335
3	0.719	10.265	75.600	0.719	10.265	75.600
4	0.600	8.571	84.171	0.600	8.571	84.171
5	0.453	6.475	90.646			
6	0.410	5.856	96.502			
7	0.245	3.498	100.000			
14	0.079	0.466	99.361			
15	0.053	0.312	99.673			
16	0.033	0.195	99.868			
17	0.023	0.132	100.000			

**Table 6:** Rotated component matrix of biomimicry drivers for sustainable transformation in the CI.

Biomimicry Drivers for Sustainable Transformation in the CI	Component
	1
Developing a legal and regulatory framework	0.782
Developing a policy monitoring system	0.766
Improving government support and intervention	0.752
Improving availability of biomimetic framework and measurement standard	0.672
Improving availability of biomimetic technology	0.637
Improved affordability of biomimetic materials	0.598
Providing biomimicry innovation and certification	0.556

Extraction Method: Principal Axis Factoring. Rotation Method: Varimax with Kaiser Normalisation ^a^; ^a^: Rotation converged in 7 iterations.

## Data Availability

The datasets employed in this study are available upon request from the corresponding author.

## Abbreviations

The following abbreviations are used in this manuscript:

CI	Construction Industry
SD	Sustainable Development
SCP	Sustainable Construction Practice
PPM	Push–Pull–Mooring
EFA	Exploratory Factor Analysis
